# Seroprevalence of SARS-CoV-2 and risk factors in Bantul Regency in March-April 2021, Yogyakarta, Indonesia

**DOI:** 10.1371/journal.pgph.0000698

**Published:** 2023-06-26

**Authors:** Riris Andono Ahmad, Citra Indriani, Risalia Reni Arisanti, Ratih Oktri Nanda, Yodi Mahendradhata, Tri Wibawa

**Affiliations:** 1 Faculty of Medicine, Public Health, Center for Tropical Medicine, Nursing Universitas Gadjah Mada, Yogyakarta, Indonesia; 2 Faculty of Medicine, Public Health, Department of Biostatistics, Epidemiology and Population Health, Nursing Universitas Gadjah Mada, Yogyakarta, Indonesia; 3 Faculty of Medicine, Public Health, Department of Health Policy and Management, Nursing Universitas Gadjah Mada, Yogyakarta, Indonesia; 4 Faculty of Medicine, Public Health, Department of Microbiology, Nursing Universitas Gadjah Mada, Yogyakarta, Indonesia; University of Embu, KENYA

## Abstract

COVID-19 case counts in Indonesia inevitably underestimate the true cumulative incidence of infection due to limited diagnostic test availability, barriers to testing accessibility and asymptomatic infections. Therefore, community-based serological data is essential for understanding the true prevalence of infections. This study aims to estimate the seroprevalence of SARS-CoV-2 infection and factors related to the seropositivity in Bantul Regency, Yogyakarta, Indonesia. A cross-sectional study involving 425 individuals in 40 clusters was conducted between March and April 2021. Participants were interviewed using an e-questionnaire developed in the Kobo toolbox to collect information on socio-demographic, COVID-19 suggestive symptoms, history of COVID-19 diagnosis and COVID-19 vaccination status. A venous blood sample was collected from each participant and tested for immunoglobulin G (Ig-G) SARS-CoV-2 antibody titers using the enzyme-linked immunosorbent assay (ELISA). Seroprevalence was 31.1% in the Bantul Regency: 34.2% in semi-urban and 29.9% in urban villages. Participants in the 55–64 age group demonstrated the highest seroprevalence (43.7%; p = 0.00), with a higher risk compared to the other age group (aOR = 3.79; 95% CI, 1.46–9.85, p<0.05). Seroprevalence in the unvaccinated participants was 29.9%. Family clusters accounted for 10.6% of the total seropositive cases. No significant difference was observed between seropositivity status, preventive actions, and mobility. Higher seroprevalence in semi-urban rather than urban areas indicates a gap in health services access. Surveillance improvement through testing, tracing, and treatment, particularly in areas with lower access to health services, and more robust implementation of health protocols are necessary.

## Introduction

**Fig 1:**
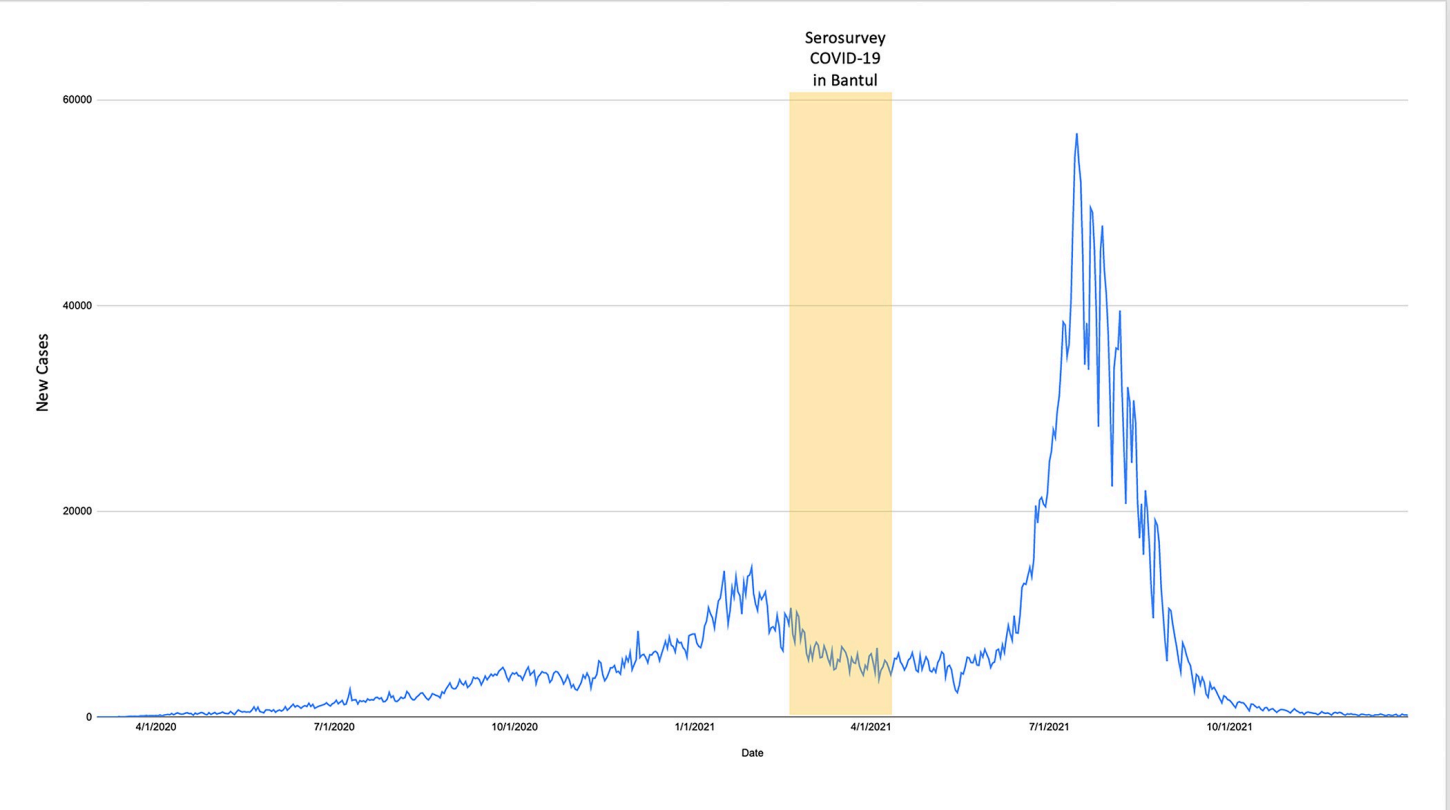
COVID-19 cases daily trend in Indonesia March 2020 –December 2021 (data source: https://ourworldindata.org/coronavirus/country/indonesia, accessed on October 4 2022).

Coronavirus disease 2019 (COVID-19) is a respiratory illness caused by the newly discovered severe acute respiratory syndrome coronavirus 2 (SARS-CoV-2), leading to a global pandemic, including Indonesia. Since the first confirmed SARS-CoV-2 on March 2 2020 [1], Indonesia has experienced exponential growth of COVID-19 cases in 34 provinces. As of May 2 2021, the government of Indonesia reported 1.677.274 confirmed COVID-19 cases and 45.796 deaths [2] and became one of the countries with high cumulative and incidence cases of COVID-19. The number of COVID-19 cases in Indonesia has been increasing rapidly since January, leading to the highest peak thus far, with provinces in Java making up 64% of the national cases. Two notable waves were observed from March 2020 to December 2021 **(Fig 1).** Based on the daily trend of COVID-19 cases, the first wave of COVID-19 cases was observed in January, with the highest number of reported cases reaching 14518 cases on January 16. After that, the cases fluctuated and reached the highest during the third wave in September 2022, with 56.757 recorded cases.

However, these case counts inevitably underestimate the true cumulative incidence of infection because of limited diagnostic test availability, barriers to testing accessibility, and asymptomatic infections, not to mention underreporting [2]. As a consequence, the national prevalence of SARS-CoV-2 remains unknown.

The lack of clarity about the number of SARS-CoV-2 infections across Indonesia limits the Indonesian government’s ability to plan appropriately, prepare and respond to this epidemic. Monitoring the incidence of newly diagnosed cases of severe COVID-19 and the case fatality rate is critical to address the demands on the healthcare system.

One of the epidemiological investigations used to determine the level of disease spread is to conduct a seroprevalence survey. According to the Centers for Disease Control and Prevention (CDC), this survey uses serological tests to detect antibodies in the blood, indicating an infection [3]. This test uses an enzyme-linked immunosorbent (ELISA) where the antigen used is purified SARS-CoV-2 S protein (without live virus). Population-based serological testing provides better estimates of the cumulative incidence of infection by complementing diagnostic testing of acute illness and helping to inform the public health response to COVID-19. A seroprevalence study could also be a powerful tool to detect subclinical infections and improve policy-making in the country [4,5]. Furthermore, as the world moves through the vaccine and variant era, synthesizing seroepidemiology findings is increasingly important to track the spread of infection, identify disproportionately affected groups, and measure progress towards herd immunity [2].

Seroprevalence varies geographically; the denser urban areas have higher seropositivity rates than rural areas [6]. A study in East Java, Indonesia, in the second semester of 2020 showed a higher prevalence in Surabaya (13.1%), which is an urban area, than in Jombang (9,9%), a rural area [7]. The epidemiological trend also implicates SARS-CoV-2 spread among rural communities only later in the epidemic wave [8,9], which would require sound anticipatory interventions. A seroprevalence survey involving more diverse groups of people among urban and rural communities is necessary to grasp the overall picture of SARS-CoV-2 infection. Bantul Regency has become one area that contributes to many cases and leads to the high transmission of COVID-19 in the Yogyakarta Provinces. Therefore, this study aims to estimate the SARS-CoV-2 seroprevalence, seropositive risk factors, and COVID-19 vaccine acceptance in Bantul Regency, Yogyakarta.

## Materials and methods

### Study setting

**Fig 2:**
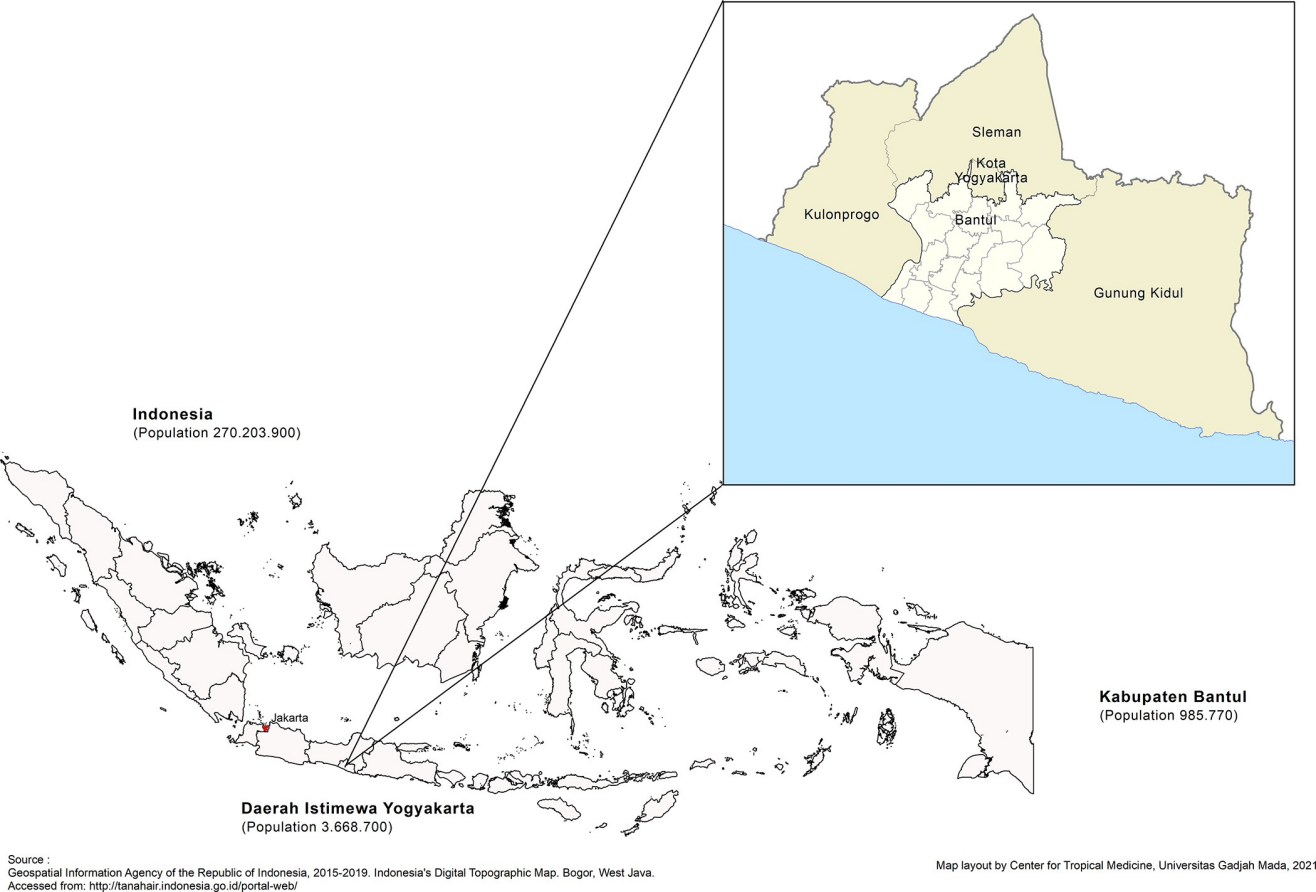
Map of Bantul Regency, Special Region of Yogyakarta, Indonesia.

Bantul Regency is located in the southern region of Yogyakarta Province, covering 506.85 km^2^ **(Fig 2).** The regency consists of 17 sub-districts and 75 villages, 30% semi-urban. Most adults are engaged in the non-formal sector, such as farming, trade and the service industry [10]. The daily mobility of residents between districts to and from Bantul is high, which may increase the risk of disease transmission [11].

### Study design and sampling

A cross-sectional study was conducted from March to April 2021. The study population consisted of individuals who lived in the Bantul regency for at least six months, with 1,018,402 inhabitants [10]. EpiInfo was used to calculate the sample size.

Sampling was determined using a multistage cluster random sample adopted from the WHO/EPI rapid survey [12]. The calculated sample size was 414, which accounted for the estimated prevalence of 20%, 6% sampling error, a significance level of 0.05 with a design effect of 2 and a non-response rate of 15%. There were 40 clusters, with 11 participants per cluster. The cluster was based on the smallest administrative area called RT (*rukun tetangga* or household group). The RT consists of approximately 50–70 households.

We used systematic random sampling to select households at the selected cluster. In the selected households, we recruited all eligible household members, i.e. anyone who lived under the same building and resided in the study area for at least six months, with a minimum age of 5 years old.

Participants must meet the following inclusion criteria: residing in the study location at least six months before the survey commences, age of 5 years old or older, able to communicate verbally, give written consent to participate in the research or consent from parents/ guardians for respondents under 18 years old. Participants aged 13–17 were interviewed by their parents/guardians. However, for participants under 13 years old, interviews were conducted with the parents. Meanwhile, the exclusion criteria include people with existing chronic illnesses (including immunocompromised individuals with a history of blood disorders and people with mental disorders).

### Data collection

A door-to-door visit was conducted to collect the primary data. Written informed consent was obtained from each study participant before data collection. Fifteen enumerators, including a phlebotomist, were involved in the data collection. Two supervisors were assigned to ensure the methodology and conduct the spot check. All study teams were involved in four training days regarding methods, data entry, phlebotomy and ethics in research.

Using an aseptic procedure, two millilitres of blood were drawn from a cubital vein with a disposable sterile syringe. The specimen was then kept in an EDTA tube. Before transportation, blood samples were placed in a cool box with an ice pack and transported within the same day to the laboratory. Blood samples were further stored in the refrigerator. Each participant’s data included a unique identifier (barcode label) linked to their blood sample and data for tracking and confidentiality. The blood sample was transported, examined and stored in the Laboratory of Microbiology, Faculty of Medicine, Public Health, and Nursing UGM. Plasma was then tested for Ig-G anti-SARS-Cov2 procedures using (Human Anti-2019 n-CoV(N) IgG ELISA Kit V1.5 FineTest [13].

Risk factors information at household and individual levels were obtained using an electronic structured questionnaire developed in the KoBo toolbox [14,15]. Variables included in the household questionnaire were socio-demographic information (age, gender, relationship with the head of household, and household income). Individual questionnaires were used to gather socio-demographic information (age, gender, highest education, occupation), COVID-19 vaccination status, previous diagnosis of COVID-19 and symptoms related to COVID-19 within the last six months, preventive actions taken, and mobility in the previous two weeks. The data manager validated data daily. A day after the data entry, the data manager sent the supervisor feedback and confirmation regarding completeness and data consistency.

Updated cumulative data on notified cases were obtained through the COVID-19 surveillance system conducted by Bantul District Health Office Bantul from March to April 2021. COVID-19 was defined as a symptomatic or asymptomatic person with a positive PCR result tested for SARS-CoV-2. Population numbers per sub-district were obtained through Bantul Statistical Bureau for calculating incidence per 1000 population [10]. In addition, data on urban-rural classification was obtained from the National Statistical Bureau [16].

### Statistical analysis

Proportion and percentage describe the seroprevalence and socio-demographic characteristics of study participants. A map developed in ArcGIS spatially compared seroprevalence and incidence of notified cases by district and urban-rural status [17,18]. Bivariate analysis was conducted to identify the association between presumed risk factors (age, gender, occupation, comorbidities, prevention taken and mobility over the last two weeks) and anti-SARS CoV-2 seropositivity. After considering collinearity, a multiple logistic regression was used to evaluate risks between seropositive and seronegative groups with adjustment for sex. The P-value would be considered statistically significant at p < 0.05. All statistical analysis was performed using STATA 14.0. Raw data used for analysis is provided in (S1 Data).

### Ethics

The Medical and Health Research Ethics Committee of the Faculty of Medicine, Public Health and Nursing Universitas Gadjah Mada approved the study (Ref: KE/1242/12/2020). Written informed consent was obtained from adult respondents and parents of enrolled children. Confidentiality of information from the respondents was upheld with utmost care throughout data collection, processing and analysis for all data collected. Therefore, their names were included in the notes only for traceability and referral during the data analysis.

## Results

### Characteristics of study participants

A total of 425 people participated and were tested during the survey. See 1 File. The number of females (59.1%) is higher than that of males (40.9%); 47.2% of the participants were unemployed/students/housewives, and 72.5% lived in urban areas. The majority of the participants had no comorbidities and were not vaccinated.

The prevalence of SARS-CoV-2 seropositivity among the participants in this study was 31.1% (n = 132/425). A significant difference was observed in the seroprevalence among age groups (p = 0.000), with the highest proportion reported in the 55–64 age group (43.7%; n = 31/71). Meanwhile, the under-15 years age group showed no seropositivity.

A significant difference was also observed among occupation groups (p = 0.009). The highest seroprevalence was demonstrated by participants working as daily workers/farmers (37.2%), followed by professional/health workers (34.6%), and unemployed/students/housewives (26%). Seroprevalence did not differ between semi-urban and urban areas, even though we observed that semi-urban areas had higher seroprevalence (34.2%; n = 40/117) than urban areas (29.9%; n = 92/308). Females demonstrated higher seroprevalence; nevertheless, no association was found between genders and seropositivity. Seropositivity also does not differ in occupation category (Table 1).

**Table 1:** Distribution of study participants by background characteristics.

Characteristic	Total	IgG-positive	IgG-negative	P-value
	n = 425	n = 132	n = 293	
**Sex, n (%)**				
Male	174 (40.9)	52 (29.9)	122 (70.1)	0.663
Female	251 (59.1)	80 (31.9)	171 (68.1)	
**Age group, years, n (%)**				
< = 14	16 (3.8)	0 (0.0)	16 (100)	< 0.001
15–24	52 (12.2)	8 (15.4)	44(84.6)	
25–34	51 (12.0)	8 (15.7)	43(84.3)	
35–44	90 (21.2)	33 (36.7)	57(63.3)	
45–54	92 (21.7)	32 (34.8)	60 (65.2)	
55–64	71 (16.7)	31 (43.7)	40 (56.3)	
65+	53 (12.5)	20 (37.7)	33 (62.3)	
**Occupation, n (%)**				
Unemployed/students/Housewives	200 (47.2)	52 (26.0)	148 (74.0)	0.009
Professional/health worker	130 (30.7)	45 (34.6)	85 (65.3)	
Daily worker/farmer	94 (22.2)	35 (37.2)	59 (62.8)	
**Residence set, n (%)**				
Urban	308 (72.5)	92 (29.9)	216 (70.1)	0.390
Semi-urban area	117 (27.5)	40 (34.2)	77 (65.8)	
**Smoking, n (%)**				
Smoker	74 (18.1)	14 (18.9)	60(81.1)	0.007
Non-smoker	335 (81.9)	118 (35.2)	217(64.8)	
**History of chronic disease, n (%)**				
Yes	96 (22.6)	37 (38.5)	59 (61.5)	0.072
No	329 (77.4)	95 (28.9)	234 (71.1)	
**Diabetes mellitus, n (%)**				
Yes	15 (3.5)	6 (40.0)	9 (60.0)	0.514
No	394 (92.5)	126 (32.0)	268 (68.0)	
**Hypertension, n (%)**				
Yes	65 (15.9)	23 (35.4)	42 (64.6)	0.559
No	344 (84.1)	109 (31.7)	235 (68.1)	
**Obesity, n (%)**				
Yes	8 (1.9)	4 (50)	4 (50)	0.279
No	401 (98.1)	128 (31.9)	273 (68.1)	
**Previous COVID-19 diagnosis, n (%)**				
Yes	3 (0.7)	1 (33.3)	2 (66.7)	1.000
No	422 (99.3)	131 (31.0)	291 (68.9)	
**COVID-19 symptoms n (%)**				
Yes	155 (36.4)	44 (28.4)	111 (71.6)	0.4
No	270 (63.4)	88 (32.6)	182 (67.4)	
**COVID-19 vaccination, n (%)**				
Yes, at least one dosage	13 (3.1)	9 (69.2)	4 (30.8)	0.005
Not yet	412 (96.9)	123 (29.9)	289 (70.2)	
**Preventive measures and mobility**				
***Wearing masks when going out***				
Always	380 (89.4)	116 (30.5)	264 (69.5)	0.491
Not always	45 (10.6)	16 (35.6)	29 (64.4)	
***Washing hands for at least 20 seconds with running water***				
Always	344 (80.9)	106 (30.8)	238 (69.2)	0.822
Not always	81 (19.1)	26 (32.1)	55 (67.9)	
***Maintain a physical distancing (1-2m) in the public area***				
Always	274 (64.5)	82 (29.9)	192 (70.1)	0.497
Not always	151 (35.5)	50 (33.1)	101 (66.9)	
**Mobility**				
***Attending invitations to traditional or religious activities (e*.*g*. *weddings*, *funerals)***				
Always	39 (9.2)	14 (35.9)	25 (64.5)	0.493
Not always	386 (90.8)	118 (30.6)	268 (69.4)	
***Visiting relatives or friends or other people due to important matters***				
Always	42 (9.9)	13 (30.9)	29 (69.1)	0.987
Not always	383 (90.1)	119 (31.1)	264 (68.9)	
***Going to the markets/shops/offices/crowds***				
Always	166 (39.1)	50 (30.1)	116 (69.9)	0.738
Not always	259 (60.9)	82 (31.7)	177 (68.3)	
***Staying at home*, *except for essential matters***				
Always	313 (73.7)	94 (30.1)	219 (69.9)	0.444
Not always	112 (26.3)	38 (33.9)	74 66.1)	

This study also explored seroprevalence based on the presence of selected chronic diseases and the history of comorbidities. Participants with a history of chronic conditions accounted for 22.6% of the total participants. Among those with comorbidities, seroprevalence for participants with obesity, diabetes mellitus, and hypertension was 50%, 40% and 35.4%, respectively.

Participants with a prior diagnosis of COVID-19 in the last six months accounted for 1 out of 132 seropositive cases. Seroprevalence among participants with no previous experience of COVID-19-related symptoms was found to be 31.1%. Seropositivity was observed in 14 participants from seven households, making family clusters account for 10.6% of the total positive cases.

Most of the participants in this study reported decreased social interactions and mobility during this period. Study participants stated that they attended fewer traditional and religious activities (90.8%), had reduced visits to relatives and friends (90.1%, decreased visits to markets (60.9%), and spent more time at home, except for essential tasks ((73.7%). If participants did leave home, they reported wearing masks (89.4%), regular washing of hands for 20 seconds with running water (80.9%) and maintaining physical distancing in public areas (64.5%). However, no significant differences were observed between individual preventive actions and the level of mobility with seropositivity status.

### Seroprevalence of anti-SARS-CoV-2 based on the geographical distribution

**Fig 3:**
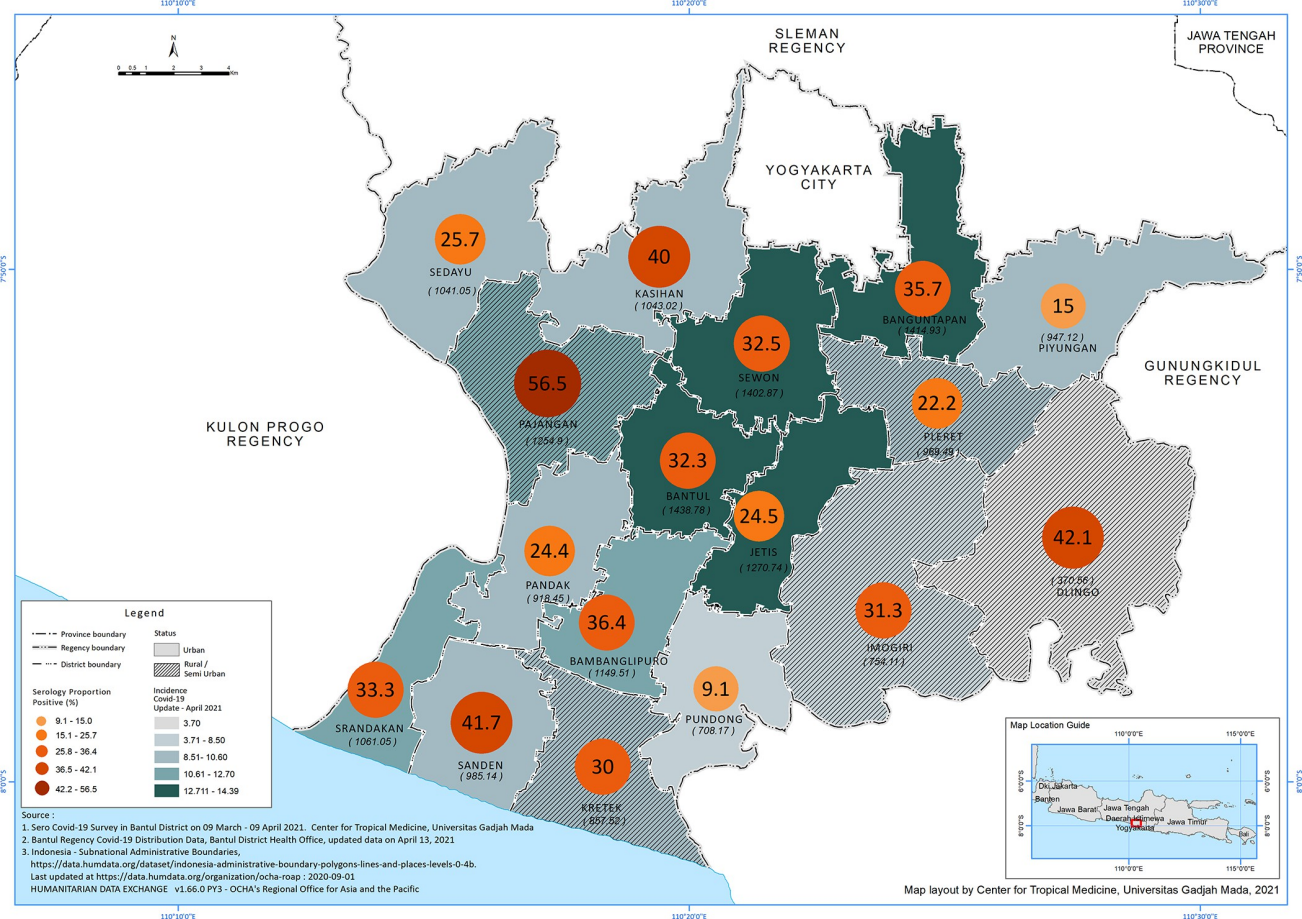
Seroprevalence and reported cumulative incidence of SARS-Cov-2 in April 2021 Based on District.

The highest seroprevalence was observed in 3 semi-urban areas (Pajangan, Dlingo, Sanden). Most semi-urban areas have relatively higher seroprevalence than urban areas. However, we found no statistical significance **(Fig 3).** The highest distribution of confirmed cases acquired from routine regional data reported higher cumulative cases in urban areas, such as Banguntapan, Bantul, Sewon, and Jetis.

### Risk factors associated with SARS-CoV-2 seropositivity

This study further explored the risk factors associated with the seropositivity of SARS-CoV-2, adjusting for age, sex, occupation, comorbidities, and vaccination status. A significant association was observed within specific age groups. The odds of SARS-CoV-2 seropositivity are higher in the age 55–64 (adjusted odds ratio [aOR] = 3.79; 95% CI 1.46–9.85, p = 0.006).

Females demonstrated higher seroprevalence. Nevertheless, no association was found between genders and seropositivity. Seropositivity also does not differ in occupation category (aOR = 0.81; 95% CI 0.50–1.31, p>0.005) (Table 2).

**Table 2:** Factors associated with SARS-Cov-2 seropositivity in Bantul Regency (N = 425).

Risk Factors	ELISA+ (n = 132)	Adjusted OR (95% CI)	*p*-value
**Age group**			
< = 14	0	NA	NA
15–24	8	Reference	NA
25–34	8	0.93 (0.31–2.79)	0.893
35–44	33	2.71 (1.08–6.81)	0.034
45–54	32	2.67 (1.05–6.80)	0.040
55–64	31	3.79 (1.46–9.85)	0.006
65+	20	3.38 (1.27–9.00)	0.015
**Sex**			
Male	52	0.81 (0.50–1.31)	0.388
Female	80	Reference	NA
**Occupation**			
Unemployed/students/Housewives	52	Reference	NA
Professional/health worker	45	1.14 (0.64–2.00)	0.661
Daily worker/farmer	35	1.27 (0.70–2.31)	0.441
**Comorbidities**			
Hypertension	23	0.87 (0.48–1.57)	0.646
Obesity	4	2.46 (0.57–10.65)	0.229
Diabetes Mellitus	6	1.12 (0.38–3.31)	0.832
**Vaccination Status**			
Yes	14	Reference	NA
No	13	0.19 (0.05–0.68)	0.010

Comorbidities were presumed as one of the risk factors of SARS-CoV-2 seropositivity. The odds of SARS-CoV-2 seropositivity were higher in participants with obesity (aOR = 2.46; 95% CI 0.57–10.65) and diabetes mellitus (aOR = 1.12; 95% CI 0.38–3.31) than participants without each respective comorbidity. However, this finding is not statistically significant (p-value>0.05).

Although vaccination had not been made available to the general public during the data collection period, most participants (91.5%) were aware that the government would provide vaccinations in the future and stated their willingness (79.1%) to be vaccinated. However, a small proportion (19.1%) said they did not want to be vaccinated (Table 3). Reasons for this included fear of adverse effects, concerns about the safety and effectiveness of the vaccines, and religious beliefs.

**Table 3:** Vaccine acceptance of the participants (N = 425).

Variables	N = 425	Percentage
***Aware that the government would provide the vaccination for the people***		
Yes	389	91.5
No	36	8.5
***Willing to get vaccinated if the government provide the vaccines***		
Yes	336	79.1
No	81	19.1
Not decided	8	1.9

## Discussion

This SARS-CoV-2 seroprevalence study found that the prevalence of IgG antibodies against SARS-CoV-2 was 31.1% in Bantul, significantly higher than the 11.4% seropositive which was found in a study in East Java in late 2020 [7]. On the other hand, surveillance data of Bantul Regency DHO reported a considerably lower cumulative incidence of 1.1% during the study. This finding is in line with previous research suggesting that the estimates obtained from seroprevalence were 18.1 times higher than the corresponding cumulative incidence of COVID-19 infections, implying that confirmed cases are a poor indicator of the extent of the disease spread [19]. While antigen testing and Polymerase Chain Reaction (PCR) to monitor COVID-19 detect the presence of specific antigens or genetic material indicating current viral infection, serological tests for COVID-19 are used to detect antibodies that the body produces in response to the virus. Serological tests can detect antibodies in people who have been infected with COVID-19 in the past, even if they were asymptomatic or had mild symptoms. Those can help identify people who may have been infected but were never diagnosed. In population-level studies, serological testing is used to estimate the proportion of people who have been infected with COVID-19 in a given area.

Seroprevalence varies geographically, and previous research indicated that urban areas mainly reported a higher number of seropositivity [6]. The reported COVID-19 cases in the Bantul regency also showed a similar trend, in which more cases were reported in urban areas (Bantul, Banguntapan, Sewon, and Jetis). However, our study showed that the three highest seropositivities were observed in semi-urban areas (Pajangan, Dlingo and Sanden). Meanwhile, areas with the highest reported COVID-19 cases showed lower seropositivity. Thus, this study suggest that the higher transmission observed in semi-urban areas was due to lower access to health services, including the lack individual testing, resulting in unreported positive cases. These discrepancies can be explained by the limited and centralized diagnosis facilities when the study was conducted.

Urban areas with dense populations were impacted earlier at the pandemic’s beginning. A previous study on epidemiological modelling predicted that COVID-19 spread faster in urban than rural areas [20]. Despite the rapid transmission, urban areas are usually supported by better surveillance management, such as more accessible testing, tracing, and infection control. Consequently, cases in urban areas were reported faster and earlier.

Meanwhile, rural areas usually have shortages in resources and workforces, which affects their ability to detect, respond, prevent, and control infectious disease outbreaks [21,22]. In this study, a large proportion of seropositivity was reported in participants with no prior diagnosis of COVID-19, indicating that the infection might be undiagnosed due to a lack of individual testing. Thus, this study suggests that the higher transmission observed in semi-urban areas was lower access to health services, including lack of individual testing, resulting in unreported positive cases.

Higher seroprevalence in this study was observed in females. However, no statistically significant gender difference was reported. A previous study also suggested that SARS-CoV-2 IgG/IgM dynamic is mainly affected by age and disease severity, not sex [23]. Higher seropositivity was observed in the age group 55–64, and this finding supports many previous studies that indicate older age as one of the risk factors for COVID-19.

This research found a portion of seropositivity among people who primarily stayed at home. Additionally, occupations requiring less mobility, such as housewives, students, and the unemployed, also accounted for seropositivity. The research also discovered a tenth of family clusters, suggesting that the transmission may have occurred at the household level. Previous research contends that the primary mode of COVID-19 infection is through the household spread [24,25].

Our study found no difference between high and low mobility, suggesting they were already widespread community transmission, even in rural areas. Preventive measures did appear to prevent acquiring an infection, which might be because it is challenging to do preventive measures in a household setting or to practice preventive measures consistently outside [26].

Surveillance and containment measures such as large-scale social restrictions and other anticipatory prevention tools become a priority to curb transmission, primarily focusing on areas with lower access to health services. Earlier in the pandemic, outbreak containment was concentrated in the dense urban areas, which may have reduced the number of cases. However, due to high mobility, the transmission slowly moved to semi-urban and rural communities. People living in rural communities might develop a false sense of security and take fewer precautions at the beginning than the urban communities [8,9].

One of the most effective ways to prevent fatalities caused by COVID-19 is through vaccination. Due to the lack of a vaccination program for general populations during the data collection period, it is reasonable that higher seroprevalence was found in unvaccinated groups. As this study explored the seroprevalence when most people were still unvaccinated, a better picture of the infection spread in Bantul was obtained since there was no implication from vaccine-induced antibodies.

Despite the findings, this study has the following limitations. Following a natural infection, antibody titers peak and begin to wane in various manners, with some in shorter duration [27]. However, this study did not consider the assay performance concerning the waning immunity. Thus it may underestimate the true prevalence. Furthermore, the analysis results only showed the association, not causality. Finally, as a cross-sectional analysis, this study only analyzed the variables at one point of time and did not explore the seroprevalence changes over a longer period. Therefore, future research is needed to conduct a periodic or longitudinal survey to determine the prevalence in the longer term.

## Conclusion

This serosurvey demonstrated a higher seroprevalence than reported data in the same period. Based on the findings, it is strongly recommended that the local government strengthen the surveillance and 3T (testing, tracing, and treatment) efforts by involving the task force at the neighbourhood community and village levels throughout Bantul Regency, particularly in areas with lower access to health services. Besides that, it needs to increase awareness and implementation of health protocols for high-mobility individuals to prevent transmission within the household. As vaccination program for the general population are being rolled out, it is crucial to provide adequate implementation information, including health resources and logistics support. This study can be implemented in other areas, both at the district/city and provincial levels, to understand the seroprevalence of SARS-CoV-2 in Indonesia better. This study provides a district-level view of the extent of COVID-19 spread and a different approach to conducting serosurvey among diverse populations in various regions to fit the gaps in understanding COVID-19’s spread globally.

## Supporting information

S1 Table The response rate of each district. (PDF)

S1 Data Data of Seroprevalence of SARS-CoV-2 in Yogyakarta. (XLSX)
