# Supplementary material for: Seroprevalence of SARS-CoV-2 and risk factors in Bantul Regency in March-April 2021, Yogyakarta, Indonesia
Source: PLOS Glob Public Health. 2023 Jun 26;3(6):e0000698. doi: 10.1371/journal.pgph.0000698 (PMC10292707; doi:10.1371/journal.pgph.0000698)
Supplement: S1 Table — (PDF) [file pgph.0000698.s001.pdf]

Response Rate of Each Districts

No	Districts	No. of Households	Visited	Visited and Agreed to Participate	Response Rate
1	Srandakan	38	12	12	100,0
2	Sanden	57	19	19	100,0
3	Kretek	26	12	12	91,7
4	Pundong	36	15	15	100,0
5	Bambangliputo	63	24	24	79,2
6	Pandak	380	102	98	92,9
7	Pajangan	52	39	39	76,9
8	Bantul	131	61	60	78,3
9	Jetis	190	93	84	88,1
10	Imogiri	139	55	53	81,1
11	Dlingo	91	76	64	40,6
12	Banguntapan	228	79	75	77,3
13	Pleret	72	13	11	90,9
14	Piyungan	119	35	34	82,4
15	Sewon	196	88	84	66,7
16	Kasihan	39	13	13	84,6
17	Sedayu	53	15	14	100,0
